# Supplementary material for: Comparative single-cell profiling reveals distinct cardiac resident macrophages essential for zebrafish heart regeneration
Source: eLife. 2023 Jul 27;12:e84679. doi: 10.7554/eLife.84679 (PMC10411971; doi:10.7554/eLife.84679)

Chromogenic *in situ* hybridization of *timp4.3* in additional zebrafish 7-dpci hearts with PBS or CL injection at 8 days before cardiac injury (-8d_PBS or -8d_CL). Dotted lines delineated injury areas (upper panels) and arrows highlight the *timp4.3* expressed cells; scale bars, 50 μm for upper panels and 10 μm for lower panels. CL, Clodronate liposomes
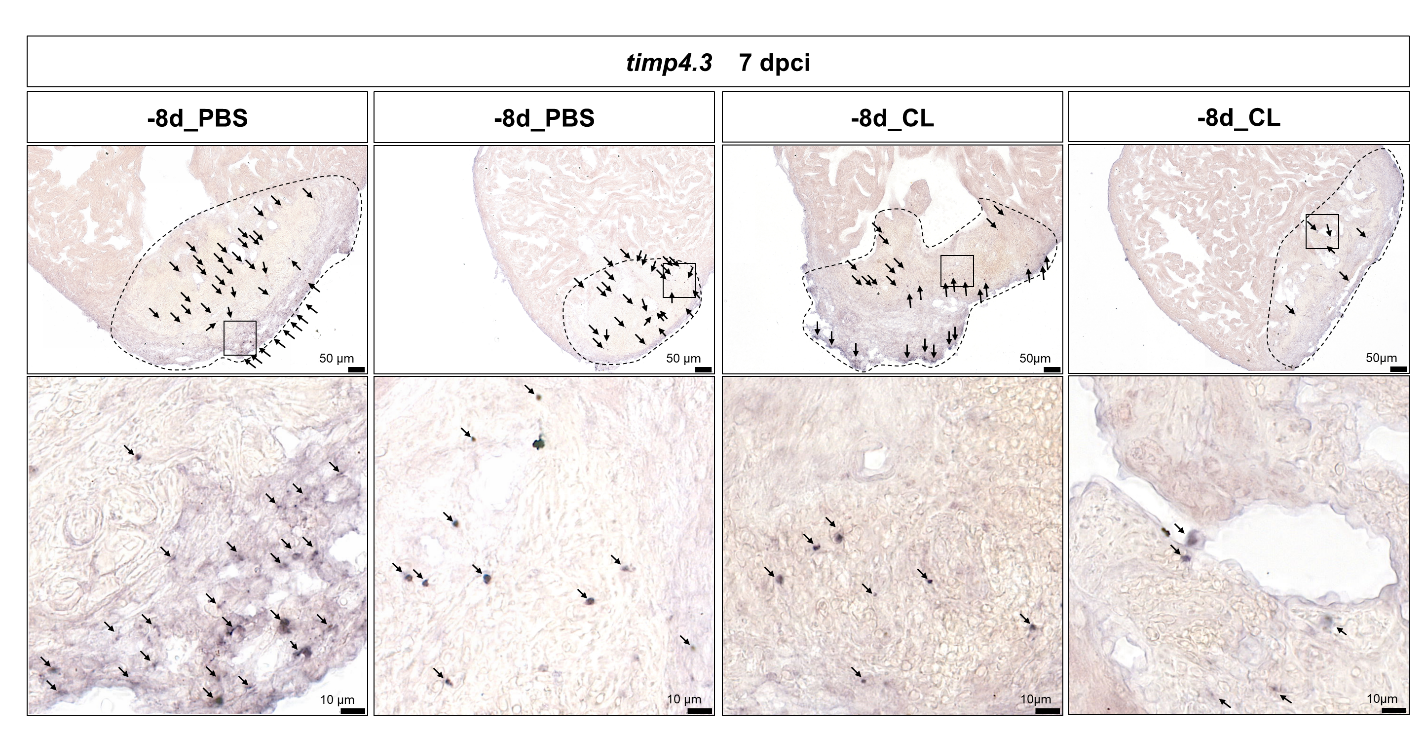

Supplement: Figure 7—source data 4. [file elife-84679-fig7-data4.docx]
