# Supplementary material for: Comparative single-cell profiling reveals distinct cardiac resident macrophages essential for zebrafish heart regeneration
Source: eLife. 2023 Jul 27;12:e84679. doi: 10.7554/eLife.84679 (PMC10411971; doi:10.7554/eLife.84679)
Supplement: MDAR checklist [file elife-84679-mdarchecklist1.pdf]

eLife

Westbrook Centre
Milton Road
Cambridge CB4 1YG, UK

P 01223 855340
W elifesciences.org
T @elife

Materials Design Analysis Reporting (MDAR) Checklist for Authors

The MDAR framework establishes a minimum set of requirements in transparent reporting mainly applicable to studies in the life sciences.

eLife asks authors to **provide detailed information within their article** to facilitate the interpretation and replication of their work. Authors can also upload supporting materials to comply with relevant reporting guidelines for health-related research (see EQUATOR Network), life science research (see the BioSharing Information Resource), or animal research (see the ARRIVE Guidelines and the STRANGE Framework; for details, see *eLife*'s Journal Policies). Where applicable, authors should refer to any relevant reporting standards materials in this form.

For all that apply, please note **where in the article** the information is provided. Please note that we also collect information about data availability and ethics in the submission form.

Materials:

Newly created materials	Indicate where provided: section/figure legend	N/A
The manuscript includes a dedicated "materials availability statement" providing transparent disclosure about availability of newly created materials including details on how materials can be accessed and describing any restrictions on access.		N/A

Antibodies	Indicate where provided: section/figure legend	N/A
For commercial reagents, provide supplier name, catalogue number and RRID, if available.	1. Mpx antibody<br>Section “Immunostaining and imaging” of Materials and Methods, line 682.<br>Cat. number: GTX128379<br>RRID: AB_2885768<br>2. Anti-mCherry antibody<br>Section “Immunostaining and imaging” of Materials and Methods, line 683.<br>Cat. number: ab205402<br>RRID: AB_2722769<br>3. Anti-HMOX1 antibody<br>Section “Immunostaining and	

	imaging" of Materials and Methods, line 683.<br>Cat. number: ARP45222_P050<br>RRID: AB_2046270<br>4. Anti- Digoxigenin Fab fragments Antibody, AP Conjugated<br>Section "*In situ* hybridization" of Materials and Methods, line 709.<br>Cat. number: 11093274910<br>RRID: AB_514497	

**DNA and RNA sequences**	**Indicate where provided: section/figure legend**	**N/A**
Short novel DNA or RNA including primers, probes: Sequences should be included or deposited in a public repository.	Yes, the oligo list with sequences was provided in "Key sources table".	

**Cell materials**	**Indicate where provided: section/figure legend**	**N/A**
Cell lines: Provide species information, strain. Provide accession number in repository OR supplier name, catalog number, clone number, OR RRID.		N/A
Primary cultures: Provide species, strain, sex of origin, genetic modification status.		N/A

**Experimental animals**	**Indicate where provided: section/figure legend**	**N/A**
Laboratory animals or Model organisms: Provide species, strain, sex, age, genetic modification status. Provide accession number in repository OR supplier name, catalog number, clone number, OR RRID.	Both male and female AB zebrafish were raised and maintained at our in-house fish facility with the animal protocol approved by the Institutional Animal Care and Use Committee of Academia Sinica (Protocol ID: 18-12-1241).<br>Section "Experimental models" of	

	Materials and Methods, line 626.	
Animal observed in or captured from the field: Provide species, sex, and age where possible.		N/A

**Plants and microbes**	**Indicate where provided: section/figure legend**	**N/A**
Plants: provide species and strain, ecotype and cultivar where relevant, unique accession number if available, and source (including location for collected wild specimens).		N/A
Microbes: provide species and strain, unique accession number if available, and source.		N/A

**Human research participants**	**Indicate where provided: section/figure legend) or state if these demographics were not collected**	**N/A**
If collected and within the bounds of privacy constraints report on age, sex, gender and ethnicity for all study participants.		N/A

Design:

**Study protocol**	**Indicate where provided: section/figure legend**	**N/A**
If the study protocol has been pre-registered, provide DOI. For clinical trials, provide the trial registration number OR cite DOI.		N/A

**Laboratory protocol**	**Indicate where provided: section/figure legend**	**N/A**
Provide DOI OR other citation details if detailed step-by-step protocols are available.		N/A

**Experimental study design (statistics details) ***		
**For in vivo studies: State whether and how the following have been done**	**Indicate where provided: section/figure legend. If it could have been done, but was not, write “not done”**	**N/A**
Sample size determination	For the bulk RNAseq, we pooled 3 hearts in one sample and prepared two samples for each time point/treatment.<br>Section “Next-generation RNA sequencing analysis”, line 722.<br><br>For the scRNAseq, we isolated and pooled large amounts of target cells from 35-45 hearts for each time point and condition. The number is in the section “Single-cell RNA-sequencing (scRNAseq) and bioinformatic analysis” of Material and Method, line 746.<br><br>For functional validation (-8d_CL/-1m_CL experiments), we collected $\geq$ 5 hearts for each time point/treatment.	
Randomisation	Male and female fish were collected randomly before the Intraperitoneal injection. Fish were maintained on the same rack in the same facility before cryoinjury. Fish were randomly taken during cryoinjury.	
Blinding		N/A
Inclusion/exclusion criteria	For scRNAseq analyses, the quality control filtering of single-cell transcriptomes is provided in the section “Single-cell RNA-sequencing (scRNAseq) and bioinformatic analysis” of Material and Method, line 746 as well as Figure 2-source data 1.	

	For functional validation, two exclusions happened in the examination of -8d_CL TUNEL assay (Figure 7D) and -1m_CL Neutrophil retention (Figure 7-figure supplement 2B), respectively. The outliers were identified by the analysis in Prism 9. Please see Figure 7-source data 1 and Figure 7-source data 3 accordingly.	

**Sample definition and in-laboratory replication**	**Indicate where provided: section/figure legend**	**N/A**
State the number of times the experiment was replicated in the laboratory.	For the -8dCL experiments, examination results of "-8d_CL revascularized density", "-8d_CL CM proliferation and CM density" came from the two replicates. Examination results of "-8d_CL TUNEL assay" and "-8d_CL neutrophil retention" came from three replicates.<br>For the -1mCL experiments, all the examination results came from one experiment.	
Define whether data describe technical or biological replicates.	For both -8dCL and -1mCL experiments, all the examination contains $\geq$ 5 hearts as biological replicates ($n \geq 5$). The quantification is the mean value calculated from three or five discontinuous sections of each heart as technical replicates.	

**Ethics**	**Indicate where provided: section/submission form**	**N/A**
Studies involving human participants: State details of authority granting ethics approval (IRB or equivalent committee(s), provide reference number for approval.		N/A

Studies involving experimental animals: State details of authority granting ethics approval (IRB or equivalent committee(s), provide reference number for approval.	The animal protocol was approved by the Institutional Animal Care and Use Committee of Academia Sinica (Protocol ID: 18-12-1241).	
Studies involving specimen and field samples: State if relevant permits obtained, provide details of authority approving study; if none were required, explain why.		N/A

**Dual Use Research of Concern (DURC)**	**Indicate where provided: section/submission form**	**N/A**
If study is subject to dual use research of concern regulations, state the authority granting approval and reference number for the regulatory approval.		N/A

Analysis:

**Attrition**	**Indicate where provided: section/figure legend**	**N/A**
Describe whether exclusion criteria were pre-established. Report if sample or data points were omitted from analysis. If yes, report if this was due to attrition or intentional exclusion and provide justification.	The exclusion criteria were not pre-established. Two exclusions happened in the examination of -8d_CL TUNEL assay (Figure 7D) and -1m_CL Neutrophil retention (Figure 7-figure supplement 2B), respectively. The outliers were identified by the analysis in Prism 9. Please see Figure 7-source data 1 and Figure 7-source data 3 accordingly.	

**Statistics**	**Indicate where provided: section/figure legend**	**N/A**
Describe statistical tests used and justify choice of tests.	All the statistical comparisons in the study are compared the means between two groups (PBS-control v.s. CL-treated). The two groups were independent, so the unpaired Student's t-test was applied. And then, the F test is	

	applied to compare the variances in whether two groups are equally sampled from populations. If there is no significant difference in F-test, we use the standard unpaired t-test. If there is a significant difference in F-test, we use the unpaired t-test with Welch's correction. The statistical detail of all the comparisons in Figure 7, Figure 7-figure supplement 2 and Figure 8 is provided in Figure 7-source data 1, Figure 7-source data 3 and Figure 8-source data 1, respectively.	

**Data availability**	**Indicate where provided: section/submission form**	**N/A**
For newly created and reused datasets, the manuscript includes a data availability statement that provides details for access (or notes restrictions on access).	We cited the previous paper of the reused dataset of 0h, 6h, 1d, 2d, 3d, and 5 dpci, which was incorporated in Figure 1B. Please see the figure legend of Figure 1B.	
When newly created datasets are publicly available, provide accession number in repository OR DOI and licensing details where available.	Publicly available at the NCBI Sequence Read Archive with Accession NO: PRJNA900299	
If reused data is publicly available provide accession number in repository OR DOI, OR URL, OR citation.	Publicly available at the NCBI Gene Expression Omnibus (accession no:GSE94617)	

**Code availability**	**Indicate where provided: section/figure legend**	**N/A**
For any computer code/software/mathematical algorithms essential for replicating the main findings of the study, whether newly generated or re-used, the manuscript includes a data availability statement that provides details for access or notes restrictions.		N/A

Where newly generated code is publicly available, provide accession number in repository, OR DOI OR URL and licensing details where available. State any restrictions on code availability or accessibility.	Publicly available at the GitHub with URL: https://github.com/petitmingchang/Cell-cell-crosstalk-prediction	
If reused code is publicly available provide accession number in repository OR DOI OR URL, OR citation.		N/A

Reporting:

The MDAR framework recommends adoption of discipline-specific guidelines, established and endorsed through community initiatives.

**Adherence to community standards**	**Indicate where provided: section/figure legend**	**N/A**
State if relevant guidelines (e.g., ICMJE, MIBBI, ARRIVE, STRANGE) have been followed, and whether a checklist (e.g., CONSORT, PRISMA, ARRIVE) is provided with the manuscript.	Yes, we followed the ARRIVE guidelines and provided the eLife_MDAR checklist with the manuscript.	

---

* We provide the following guidance regarding transparent reporting and statistics; we also refer authors to Ten common statistical mistakes to watch out for when writing or reviewing a manuscript.

Sample-size estimation

- You should state whether an appropriate sample size was computed when the study was being designed
- You should state the statistical method of sample size computation and any required assumptions
- If no explicit power analysis was used, you should describe how you decided what sample (replicate) size (number) to use

Replicates

- You should report how often each experiment was performed
- You should include a definition of biological versus technical replication
- The data obtained should be provided and sufficient information should be provided to indicate the number of independent biological and/or technical replicates
- If you encountered any outliers, you should describe how these were handled
- Criteria for exclusion/inclusion of data should be clearly stated
- High-throughput sequence data should be uploaded before submission, with a private link for reviewers provided (these are available from both GEO and ArrayExpress)

Statistical reporting

- Statistical analysis methods should be described and justified
- Raw data should be presented in figures whenever informative to do so (typically when N per group is less than 10)
- For each experiment, you should identify the statistical tests used, exact values of N, definitions of center, methods of multiple test correction, and dispersion and precision measures (e.g., mean,

median, SD, SEM, confidence intervals; and, for the major substantive results, a measure of effect size (e.g., Pearson's r, Cohen's d)
- Report exact p-values wherever possible alongside the summary statistics and 95% confidence intervals. These should be reported for all key questions and not only when the p-value is less than 0.05.

Group allocation
- Indicate how samples were allocated into experimental groups (in the case of clinical studies, please specify allocation to treatment method); if randomization was used, please also state if restricted randomization was applied
- Indicate if masking was used during group allocation, data collection and/or data analysis
